# CircRAB11FIP1 promoted autophagy flux of ovarian cancer through DSC1 and miR-129

**DOI:** 10.1038/s41419-021-03486-1

**Published:** 2021-02-26

**Authors:** Zhanqin Zhang, Hongtao Zhu, Jianguo Hu

**Affiliations:** 1 Department of Anesthesiology & Center for Brain Science, The First Affiliated Hospital of Xi’an Jiaotong University, Xi’an, China; 2 Department of Obstetrics and Gynecology, Second Affiliated Hospital, Chongqing Medical University, Chongqing, China

**Keywords:** Cancer models, Autophagy

## Abstract

At present, no systematic and in-depth study is available on the function and potential mechanisms of circular RNA in autophagy. This study aimed to screen the expression profiles of circRNA, miRNA, and mRNA of ovarian cancer cells induced by Torin 1 (10 µM). The expression profiles of circRNA, miRNA, and mRNA were analyzed with next-generation sequencing technology. CircRAB11FIP1 expression was elevated in epithelial ovarian cancer (EOC) tissues than in normal ovarian tissues. Silencing circRAB11FIP1 inhibited the autophagic flux of ovarian cancer SKOV3 cells. However, circRAB11FIP1 overexpression activated the autophagic flux of ovarian cancer A2780 cells. CircRAB11FIP1-induced autophagy accelerated EOC proliferation and invasion. Also, circRAB11FIP1 directly bound to miR-129 and regulated its targets ATG7 and ATG14. CircRAB11FIP1 bound to desmocollin 1to facilitate its interaction with ATG101. Also, circRAB11FIP1 directly bound to the mRNA of fat mass and obesity-associated protein and promoted its expression. Then, circRAB11FIP1 mediated mRNA expression levels of ATG5 and ATG7 depending on m6A. In general, this study demonstrated that circRAB11FIP1 regulated ATG7 and ATG14 by sponging miR-129. The data suggested that circRAB11FIP1 might serve as a candidate biomarker for EOC diagnosis and treatment.

## Introduction

Circular RNA is a novel type of noncoding RNA discovered in recent years^1^. Circular RNA has various biological effects, including the regulation of cell proliferation, invasion, and apoptosis^2^. Autophagy is related to physiological states as well as a variety of pathological states, such as tumors, inflammation, and so forth^3^. Some studies confirmed the function of circular RNA in autophagy. CircHIPK2 participates in astrocyte activation by regulating endoplasmic reticulum stress and autophagy via sponging miR-124-2HG^4^. CircDnmt1 promoted the proliferation and survival of breast carcinoma cells through the activation of autophagy. CircDnmt1 directly interacted with p53 along with AUF1 to promote their nuclear translocation^5^. CircHECTD1 promoted TIPARP transcription and expression, thus activating autophagy and astrocytes^6^.

Previous studies demonstrate that circular RNA played a certain role in autophagy. At present, no systematic and in-depth study exists on the function and potential mechanisms of circular RNA in autophagy. Therefore, Torin 1-induced autophagy and sequencing were used in this study to explore the expression pattern of circular RNA in autophagy in ovarian cancer SKOV3 cells. The study also investigated the function and potential mechanism of circular RNA in autophagy.

## Materials and methods

### Ovarian tissues

Ovarian cancer tissues and serum samples from 70 epithelial ovarian cancer (EOC) and 30 matched non-carcinoma tissue samples were obtained from patients with ovarian cancer treated surgically in our hospital. The study was approved by the ethics committee of Chongqing Medical University. The pathological examination of ovarian cancer was confirmed by three pathologists.

### RNA-sequencing analysis

In this study, library establishment, data analysis, and sequencing were completed in Shanghai Lifegenes Technology Co., Ltd. Specific steps are described in detail in a previous study^7^.

### Cell culture and lentivirus transfection

In this study, the ovarian cancer cell line was cultivated with Rosewell Park Memorial Institute 1640 (Sigma-Aldrich, R8758) supplemented with 10% fetal bovine serum and streptomycin, followed by incubation at 37°C in the presence of 5% CO_2_. The lentiviral vectors (LV2-1 and LV2-2) and the lentiviral overexpression vector (LV6, all from GenePharma) were transfected with circRAB11FIP1. MIR-129 mimics and inhibitors were also provided by GenePharma. An ATG5 or ATG7 overexpression plasmid was constructed using a pCMV5 vector. The siRNA sequence targeting circRAB11FIP1 was as follows: LV2-1: 5′-CAUGAUGAAGGUGGUAUAATT-3′; LV2-2: 5′-GAUGAAGGUAUAAGUUTT′; ATG7: 5′-CAGAAGGAGUCACAGCUCUUCCUUA-3′; ATG5: 5′-CAAUCCCAUCCAGAGUUGCUUGUGA-3′; and negative control (NC) siRNA: 5′-UUCUUCGAAGGUGUCACGUTT-3′

### RIP assay

A biotin-labeled circRAB11FIP1 probe was provided by GenePharma. The procedure was performed as described previously^8^. Then, qPCR was used to determine miRNA expression.

### CircRNA pulldown

The circRNA pulldown was performed as previously described^9^. In brief, sodium dodecyl sulfate–polyacrylamide gel electrophoresis, silver staining, and MALDI-TOF-MS were performed sequentially, followed by protein identification.

### qPCR

A total RNA Rapid Extraction Kit (Bioteke Corporation, RP1201) was used for isolating the RNA from ovarian cancer cells. Then, RT-qPCR was performed using an iSCRIPT cDNA synthesis kit and an All-in-One qPCR mix kit. The circRNA primers were synthesized by Guangzhou Huiyuanyuan Pharmaceutical Technology Co., Ltd.

### Western blot analysis

The protein expression of ATG7, LC3, ATG14, and GAPDH was examined through western blot analysis. Primary antibodies (all from Abcam), including anti-ATG7 rabbit monoclonal antibody (ab133528), anti-LC3 rabbit polyclonal antibody (Sigma-Aldrich, L7543), anti-desmocollin-1 (DSC1) rabbit monoclonal antibody (ab83271), anti-ATG14 rabbit polyclonal antibody (ab227849), and anti-GAPDH rabbit monoclonal antibody (ab181602), were used in this study.

#### Co-immunoprecipitation assays

SKOV3 cells infected with LV2-NC or LV2-1 and A2780 cells infected with LV6-NC or LV6 were used for Co-IP assays. The cells were lysed in 150 mM NaCl, 20 mM Tris, and 1 mM ethylenediaminetetraacetic acid buffer containing protease and phosphatase inhibitors after 72 h and centrifuged, and the protein concentration was estimated. The lysates were precleared with protein A/G beads and then immunoprecipitated overnight with 2 µg anti-DSC1 mouse monoclonal antibody (sc-398590, Santa Cruz Biotechnology) at 4 °C. Immunoprecipitates were captured with protein A/G beads and washed extensively with NTEN buffer before elution by heating in 2× loading buffer. NTEN buffer alone was used as an NC. The eluates and reserved lysate (input) were electrophoresed, transferred to polyvinylidene difluoride membranes, and probed with anti-DSC1 mouse monoclonal antibody(sc-398590, Santa Cruz Biotechnology) and monoclonal anti-ATG101 antibody (SAB4200506, Sigma-Aldrich).

### EdU assay

A Cell-Light Apollo567 In Vitro Kit (RiboBio, Guangzhou, China) was used to assess the cell proliferation ability. Specific steps are described in detail in a previous study^7^.

### Matrigel invasion assays

The matrigel invasion assay was performed to determine the cellular invasion capacity as previously described^10^.

### Fluorescence in situ hybridization assay

The Cy3-labeled anti- circRAB11FIP1 probe, along with the fluorescein isothiocyanate-labeled anti-miR-129 probe, was provided by GenePharma. Specific steps are described in detail in a previous study^7^.

### Mouse xenograft model

The animal model was constructed as described previously^10^. The animal experiment protocol was approved by the Committee on the Use and Care of Animals (Chongqing Medical University, Chongqing, China). The SKOV3 ovarian cancer cell line was transfected with LV2-1 or LV2-NC. Thereafter, BALB/c nude mice (6-week old) were intraperitoneally injected with SKOV3 cells. The mice were killed after 5 weeks, and the number of ascites was determined.

### Statistical analysis

SPSS21.0 was used to perform statistical analyses through analysis of variance or Student’s *t* test. All values were expressed as mean±standard deviation (SD).

## Results

### circRNA expression profile was identified in SKOV3 cells

**Fig. 1:**
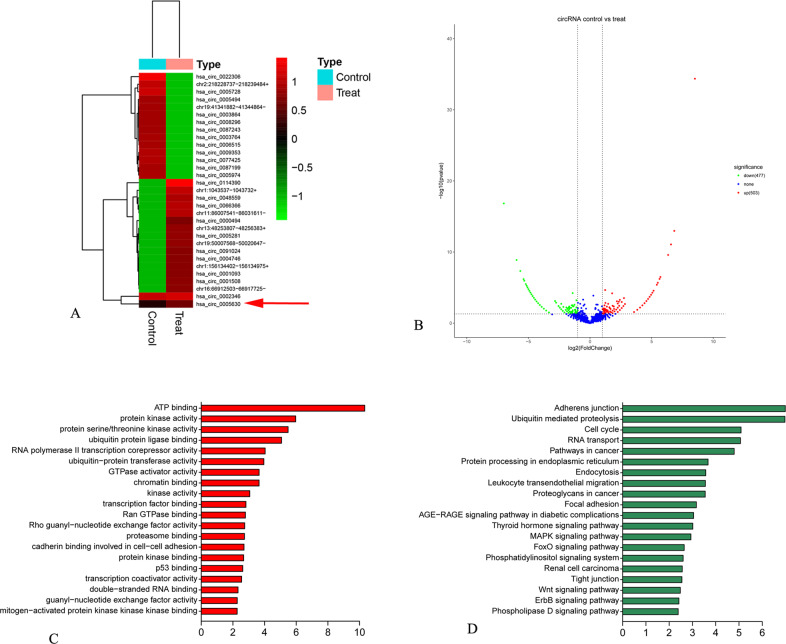
Discovery of differential circRNAs in Torin 1-treated SKOV3 cells. **A** Hierarchical clustering analysis on differential circRNAs between Torin 1 treatment (10 µM) and controls. Heatmaps were plotted according to the 30 most significant differential circRNAs. The “red” and “green” colors suggest upregulation and downregulation, respectively. **B** Volcano map for the 30 most significant differential circRNAs. **C** Functional analysis for the 30 most significant deferential circRNAs. **D** KEGG pathway enrichment analysis for the 30 most significant differential circRNAs.

The circRNA expression profile of Torin 1 (10 µM)-induced SKOV3 cells was identified via RNA-sequencing analysis. A total of 11,031 different candidate circRNAs were identified from all samples, including 504 upregulated and 478 downregulated ones. In addition, 4223 upregulated and 3952 downregulated mRNAs, along with 353 upregulated and 111 downregulated miRNAs, were detected (Fig. 1A).

The variations in circRNA expression between Torin 1-treated SKOV3 cells and NC (SKOV3 cells mixed with DMSO) were assessed using scatter plot (Fig. 1B). GO analysis found that these differentially expressed circRNAs participated in many cellular processes, including microtubule binding, actin filament binding, GTPase activator activity, and activities of activators for protein serine/threonine kinase (Fig. 1C). The pathway analysis found that the differentially expressed circRNAs participated in TGF-β signal transduction pathway, Wnt signal transduction pathway, Rap1 signal transduction pathway, Ras signal transduction pathway, and so on (Fig. 1D).

### CircRAB11FIP1 (hsa_circ_0005630) mediated the autophagic flux of EOC

**Fig. 2:**
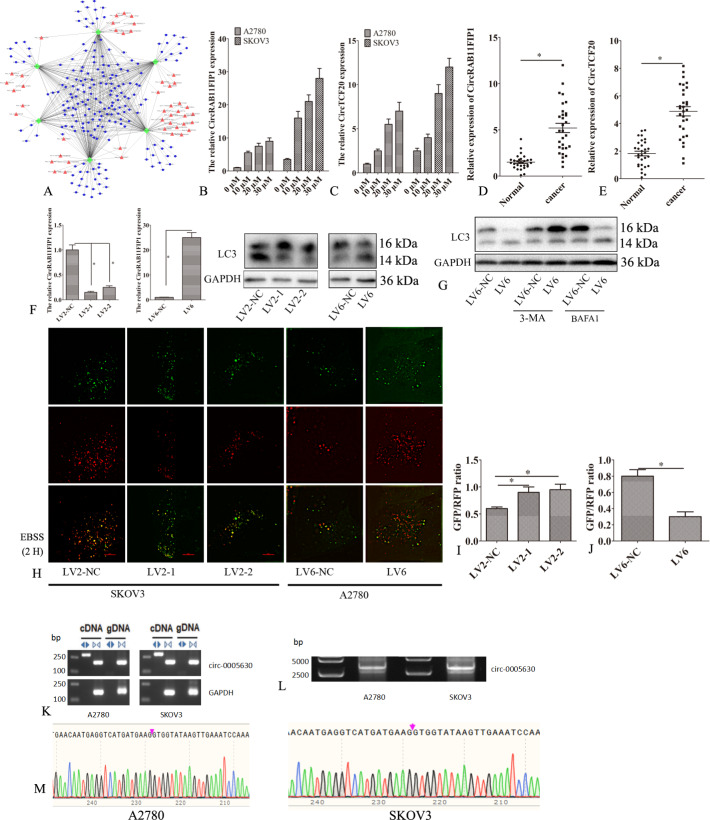
CircRAB11FIP1 (hsa_circ_0005630) regulated the autophagic flux of epithelial ovarian cancer cells. **A** Regulating network analysis among circRNAs, miRNAs, and mRNAs. **B**, **C** Torin 1 was used to treat the SKOV3 and A2780 cell lines. qPCR was conducted to detect circRNA 24 h later. **D** CircRAB11FIP1 expression levels of 30 pairs of epithelial ovarian cancer and non-carcinoma tissue samples. **E** CircRAB11FIP1 expression levels in serum samples from 70 epithelial ovarian cancer and 30 matched non-carcinoma tissue samples. **F** CircRAB11FIP1 expression levels were detected by qPCR. The LC3 level was measured by western blot analysis following circRAB11FIP1 silencing in SKOV3 cells, together with circRAB11FIP1 ectopic expression in A2780 cells. **G** LV6-NC or LV6 was transfected into A2780 cells, and 3-MA or BAFA1 was added. western blot analysis was conducted to measure the LC3 level 48 h later. **H**–**J** LV2-1, LV2-2, or LV2-NC was transfected into SKOV3 cells. LV6 or LV6-NC was transfected into A2780 cells. Later, confocal microscopy was performed to analyze the distribution of mRFP-GFP-LC3. **K** RT-PCR was performed to detect specific circRNAs in SKOV3 and A2780 cells using convergent or divergent primers. Bp, base pair, a size marker. **L** RT-PCR was carried out to amplify the full-length has_circRNA_005630 (circRAB11FIP1) in A2780 and SKOV3 cells, whereas AGE was used to confirm these amplified products. **M** Sanger sequencing was performed to verify the head-to-tail splicing of circRAB11FIP1. Error bar stands for standard error. ^*^P < 0.05. Scale bar = 5 µm.

Based on the circRNA, mRNA, and miRNA results, a regulatory network between circRNA, miRNA, and mRNA was constructed (Fig. 2A and Fig. S1). MiR-219a, miR-129, miR-146a, miR-23a, and miR-130b have been proven to regulate autophagy. CircRAB11FIP1 (hsa_circ_0005630) may potentially bind to miR-219a, miR-146a, miR-23a, and miR-129. CircTCF20 (hsa_circ_0008561) may potentially bind to miR-23a, miR-130b, and miR-129-5p. Therefore, this study investigated whether circRAB11FIP1 and circTCF20 were involved in autophagy. First, the expression of circRAB11FIP1 and circTCF20 was detected by qPCR in ovarian cancer SKOV3 and A2780 cells. Torin 1 induced the expression of circRAB11FIP1 and circTCF20 in SKOV3 and A2780 cells. The higher the concentration of Torin 1, the higher the expression of circRAB11FIP1 and circTCF20 (Fig. 2B, C). The expression of circRAB11FIP1 and circTCF20 was higher in SKOV3 cells than in A2780 cells (Fig. 2B, C). Also, the expression of circRAB11FIP1 and circTCF20 was detected in 30 EOC and 30 matched non-carcinoma tissue samples. The expression of circRAB11FIP1 and circTCF20 increased compared with that in normal ovarian tissues (Fig. 2D, E).

CircRAB11FIP1 and circTCF20 were silenced in SKOV3 cells and overexpressed in A2780 cells to determine their function. Silencing circRAB11FIP1 reduced the expression of circRAB11FIP1 and LC3-II. However, no significant change in LC3-II expression was observed after silencing circTCF20 (data not shown). On the contrary, circRAB11FIP1 overexpression promoted the expression of circRAB11FIP1 and LC3-II (Fig. 2F and Fig. S2A). LC3-II was observed owing to an increase in autophagosomes or inhibition of the autophagic flux. The circRAB11FIP1 ectopic expression promoted LC3-II accumulation. This effect was partly inhibited after adding 3-MA but not BafA1 (Figs. 2G and S2B).

**Fig. 3:**
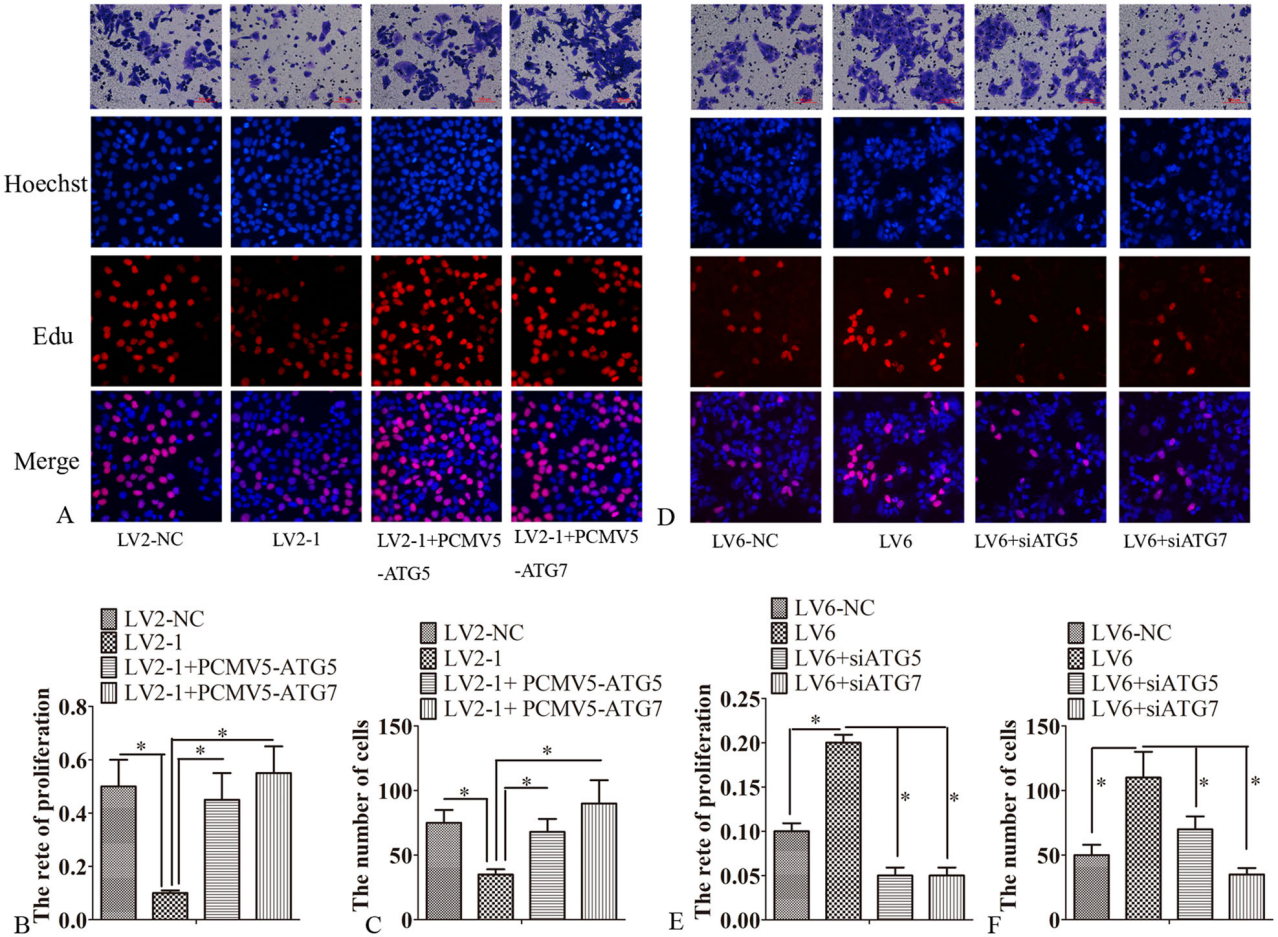
CircRAB11FIP1-induced autophagy enhanced ovarian cancer cell growth and migration. **A**–**C** LV2-1, LV2-NC, LV2-1 + PCMV5-ATG7, or LV2-1 + PCMV5-ATG5 was transfected into SKOV3 cells. Cell proliferation and migration were examined 48 h later. **D**–**F** LV6, LV6-NC, LV6 + ATG7 siRNA, or LV6 + ATG5 siRNA was transfected into A2780 cells for 48 h. Then, cell proliferation and migration were examined. Error bar stands for standard error. ^*^P < 0.05. Scale bar = 100 µm.

Furthermore, mRFP-GFP-LC3 reporter was adopted for determining the autophagic flux. Silencing circRAB11FIP1 reduced the number of red puncta in SKOV3 cells. However, circRAB11FIP1 ectopic expression increased the number of red puncta in A2780 cells, thereby indicating that circRAB11FIP1 knockdown suppressed the autophagic flux (Fig. 2H–J and Fig. S2C–S2F). Divergent primers were employed for amplifying circRNAs generated through the head-to-tail splicing. Meanwhile, circRAB11FIP1 was examined for its presence or absence and was discovered from A2780 and SKOV3 cells (Fig. 2K). Afterward, more divergent primers with partial overlapping of the 5′-end nucleotides were prepared for identifying full-length circRNAs. As suggested by RT-PCR and Sanger sequencing, circRAB11FIP1 (hsa_circRNA_005630) with stable expression was successfully amplified (Figs. 2L, 3M).

### CircRAB11FIP1-induced autophagy accelerated the proliferation and migration of SKOV3 and A2780 cells

CircRAB11FIP1 promoted autophagic flux. Therefore, the study explored whether circRAB11FIP1-induced autophagy participated in ovarian cancer cell proliferation and invasion. SKOV3 cells transfected with LV2-1 had decreased proliferation and invasion capacities. However, this effect was partly reversed after the ectopic expression of ATG5 and ATG7 (Fig. 3A–C). On the contrary, circRAB11FIP1 ectopic expression enhanced A2780 cell proliferation and invasion abilities. However, these effects were partly retarded after ATG5 or Beclin1 silencing (Fig. 3D–F).

### CircRAB11FIP1 sponging for miR-129

**Fig. 4:**
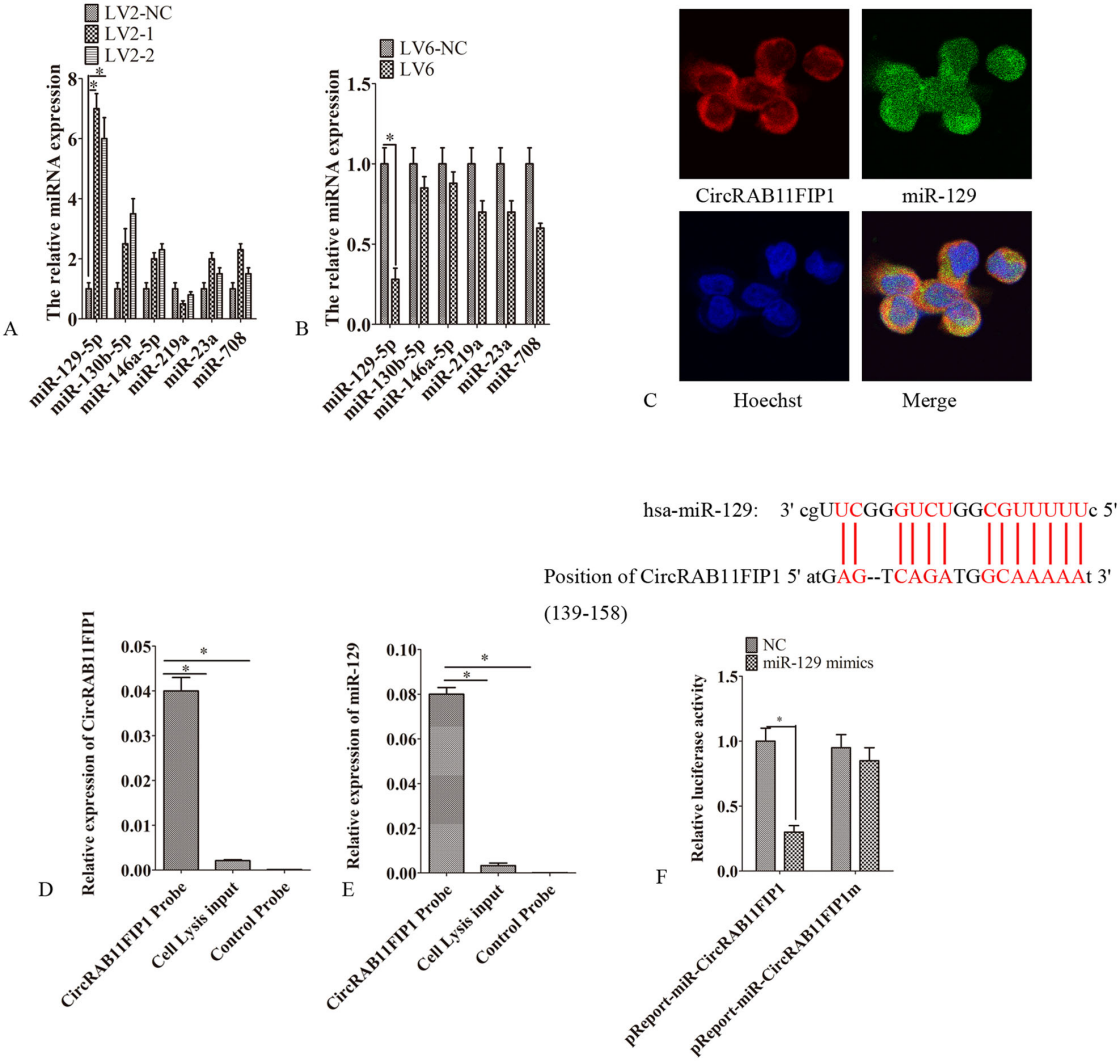
CircRAB11FIP1 sponged miR-129. **A** LV2-1, LV2-2, or LV2-NC was transfected into SKOV3 cells. Later, qPCR was performed to detect miRNA expression. **B** LV6 or LV6-NC was transfected into A2780 cells. Later, qPCR was conducted to detect miRNA expression. **C** miR-129 together with circRAB11FIP1 positions in SKOV3 cells was determined by the FISH assay. **D** CircRAB11FIP1 expression decreased in SKOV3 cell lysate, which was enriched by the circRAB11FIP1 probe, as examined using qPCR. **E** miR-129 expression decreased, which was enriched by the circRAB11FIP1 probe, as verified through qPCR. **F** Prediction of possible binding sites in circRAB11FIP1 for miR-129. SKOV3 cells were transfected with miR-129 mimic or NC (NC) RNA together with pMIR-circRAB11FIP1 or pMIR-circRAB11FIP1m, respectively. Thereafter, the luciferase activities were examined. Error bar stands for standard error. ^*^P < 0.05.

It was predicted that circRAB11FIP1 might bind to some miRNAs, including miR-129-5p, miR-130b-5p, miR-146a-5p, miR-219a, miR-23a, and miR-708 (miranda v3.3a). The levels of miR-23a, miR-130b-5p, miR-146a-5p, miR-708, and miR-219a were measured. As a result, circRAB11FIP1 silencing remarkably upregulated miR-129 expression (Fig. 4A). Besides, the circRAB11FIP1 ectopic expression notably upregulated the miR-129 level (Fig. 4B). Moreover, as revealed by the fluorescence in situ hybridization assay, circRAB11FIP1 was primarily located in the cytoplasm and overlapped with miR-129 (Fig. 4C). The RNA knockdown test showed that circRAB11FIP1 combined with miR-129 directly (Fig. 4D, E). The dual-luciferase reporter assay further confirmed this interaction. Co-transfection of circRAB11FIP1 wild-type vector and miR-129 mimics into SKOV3 cells resulted in significantly inhibited relative luciferase activity. This result suggested that circRAB11FIP1 interacted directly with miR-129 (Fig. 4F).

### CircRAB11FIP1 regulated ATG14 and ATG7 by sponging miR-129

**Fig. 5:**
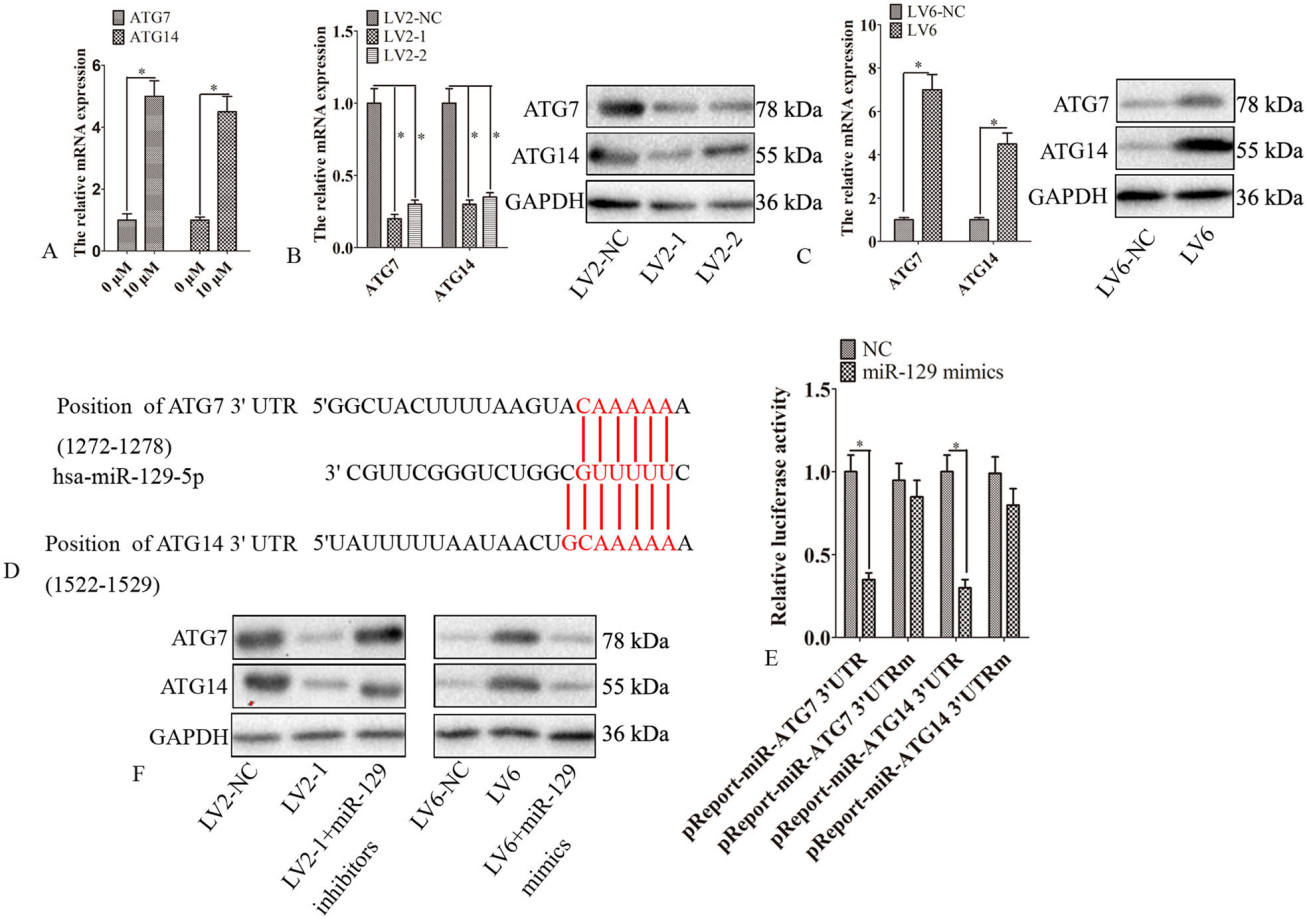
CircRAB11FIP1 mediated the expression of ATG7 and ATG14 through sponging miR-129. **A** SKOV3 cells were subjected to 24-h Torin 1 treatment. Afterward, the mRNA expression of ATG7 and ATG14 was determined using qPCR. **B** LV2-1, LV2-2, or LV2-NC was transfected into SKOV3 cells. Then, the expression of ATG7 or ATG14 was examined. **C** LV6 or LV6-NC was transfected into A2780 cells. Later, the expression of ATG7 or ATG14 was examined. **D** TargetScan was employed to predict the possible binding sites among miR-129, ATG7, and ATG14. **E** SKOV3 cells were infected with miR-129 mimic or negative control (NC) RNA along with the mutant (pMIR-ATG7 3′-UTRm and pMIR-ATG14 3′-UTRm) or wild-type (pMIR-ATG7 3′-UTR and pMIR-ATG14 3′-UTR) luciferase reporter plasmid. The luciferase activity was detected after 24 h. **F** SKOV3 cells were infected with LV2-1, LV2-NC, or LV2-1+miR-129 inhibitor. A2780 cells were infected with LV6, LV6-NC, or LV6 + miR-129 mimics. Later, the expression of ATG7 or ATG14 was examined by Western blot analysis. Error bar stands for standard error. ^*^P < 0.05; ^**^P < 0.01.

The regulatory network between circRNA, miRNA, and mRNA indicated that circRAB11FIP1 might regulate the expression of ATG14 and ATG7 through miR-129 (Fig. 2A). The sequencing data found that the mRNA expression of ATG14 and ATG7 was induced by Torn1. The study confirmed using qPCR that Torn1 induced the expression of ATG14 and ATG7 (Fig. 5A). The knockdown of circRAB11FIP1 obviously suppressed the expression of ATG7 and ATG14 within SKOV3 cells (Fig. 5B). In contrast, circRAB11FIP1 ectopic expression significantly promoted the expression of ATG7 and ATG14 within A2780 cells (Fig. 5C). It was predicted that ATG14 and ATG7 were also the targets of miR-129 (Fig. 5D). The dual-luciferase reporter assay further confirmed that ATG14 and ATG7 were the direct targets of miR-129 (Fig. 5E). The miR-129 mimics inhibited the expression of ATG14 and ATG7 in SKOV3 cells. This regulation was rescued via circRAB11FIP1 overexpression. The miR-129 inhibitor significantly elevated the expression of ATG7 and ATG14 in A2780 cells. This regulation was rescued via the knockdown of circRAB11FIP1 (Fig. 5F).

### CircRAB11FIP1 promoted autophagic flux depending on ATG14 and ATG7

**Fig. 6:**
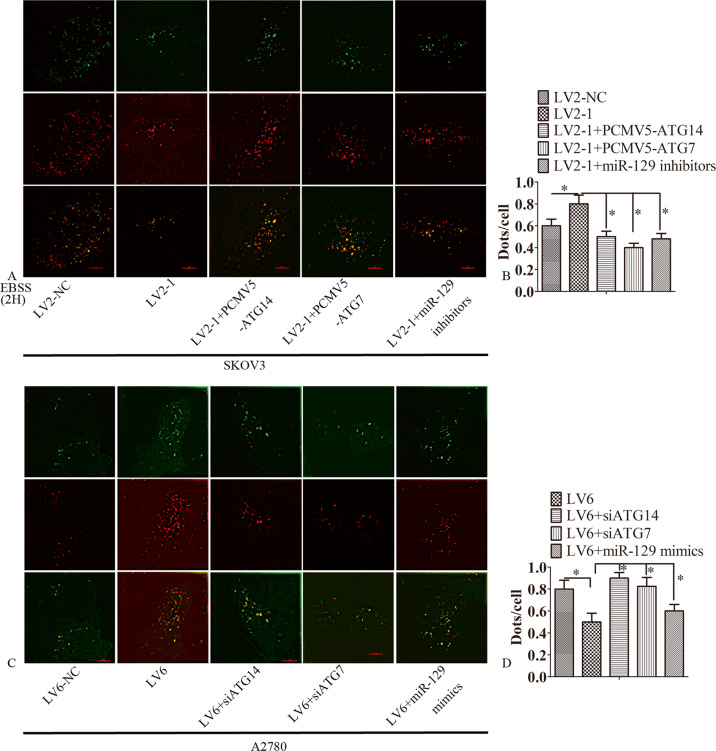
CircRAB11FIP1 promoted autophagic flux dependent on ATG14 and ATG7. **A**, **B** LV2-NC, LV2-1, LV2-1 + PCMV5-ATG14, LV2-1 + PCMV5-ATG7, or LV2-1+miR-129 inhibitor was transfected into SKOV3 cells. Then, confocal microscopy was performed to analyze the distribution of mRFP-GFP-LC3 in SKOV3 cells. **C**–**D** LV6-NC, LV6, LV6 + siATG14, LV6 + siATG7, or LV6 + miR-129 mimics was transfected into A2780 cells. Thereafter, confocal microscopy was conducted to analyze the distribution of mRFP-GFP-LC3 in A2780 cells. The Image-Pro Plus 6.0 was used to quantify LC3 dots. Each experiment was performed in triplicate, and typical results were displayed. Error bar represents standard error. ^*^P < 0.05; ^**^P < 0.01^.^ Scale bar = 5 µm.

Silencing circRAB11FIP1 suppressed the autophagic flux of SKOV3 cells. However, the overexpression of ATG14 or ATG7, or transfection of SKOV3 cells with miR-129 inhibitors, reversed this phenomenon (Fig. 6A, B). CircRAB11FIP1 ectopic expression enhanced the autophagic flux in A2780 cells; however, the silencing of ATG14 or ATG7, or transfection with miR-129 mimics, reversed this phenomenon (Fig. 6C, D).

### CircRAB11FIP1 directly bound to DSC1 protein

**Fig. 7:**
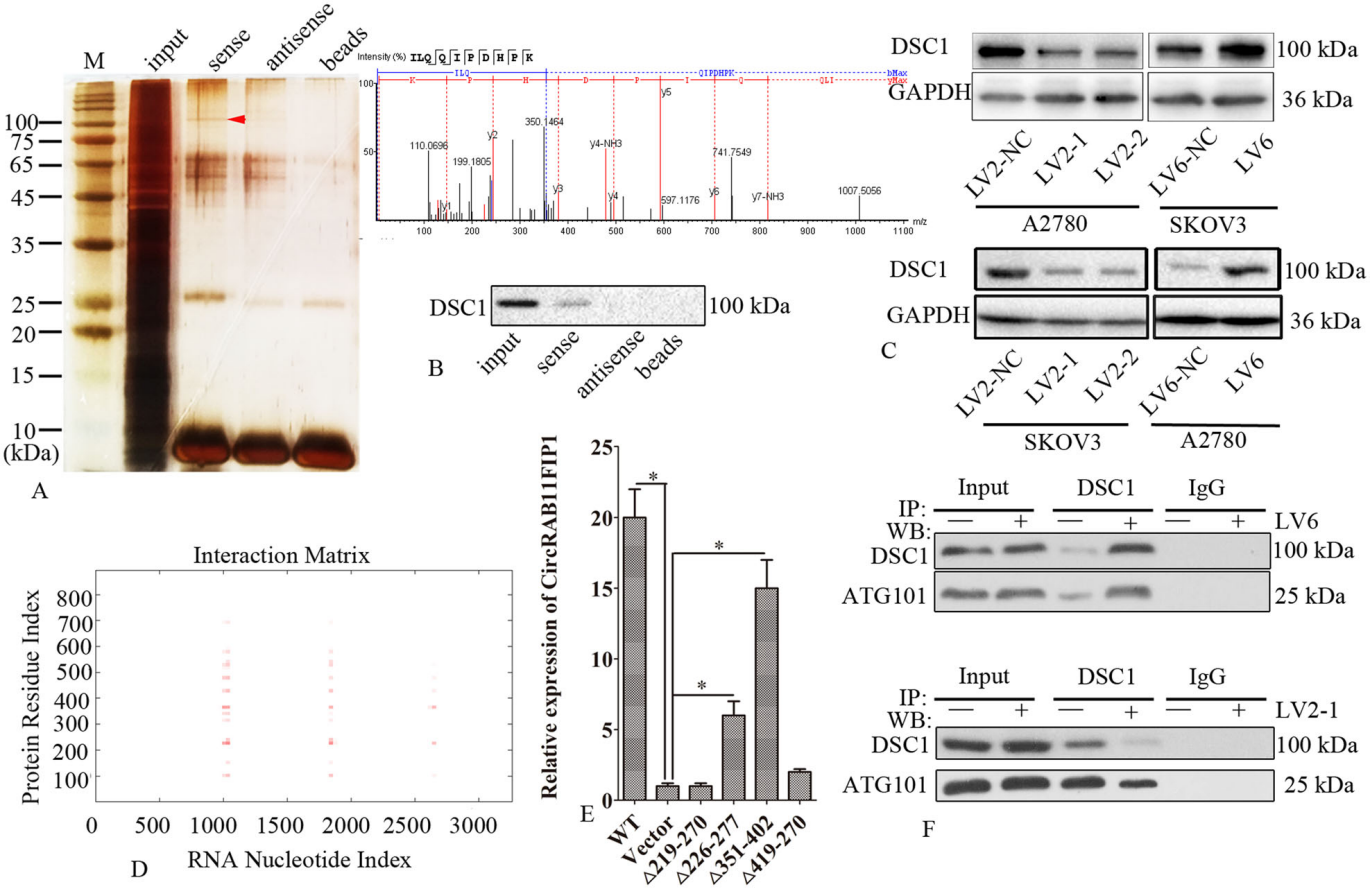
CircRAB11FIP1 directly bound to DSC1 protein. **A** Silver staining of protein gels acquired through circRAB11FIP1 RNA knockdown. **B** RNA knockdown was applied to identify circRAB11FIP1-specific binding protein gels. **C** LV2, LV2-1, or LV2-2 was transfected into SKOV3 and A2780 cells. LV6-NC or LV6 was transfected into SKOV3 and A2780 cells. Later, the DSC1 protein level was detected through western blot analysis. **D** catRAPID was used to predict possible binding sites between circRAB11FIP1 and DSC1 protein. **E** DSC1 DNA fragments were cloned to the PCMV5 plasmid. PCMV5 vector, PCMV5-DSC1, PCMV5-Δ26-77, PCMV5-Δ139-190, or PCMV5-Δ176-227, PCMV5-Δ239-290 was transfected into SKOV3 cells. PCR was conducted to detect circRAB11FIP1 expression after 48 h. **F** Co-IP and western blot assays showing the interaction between DSC1 and ATG101 in SKOV3 and A2780 cells stably transfected with LV6 or LV2-1, respectively. Error bar stands for standard error. ^*^P < 0.05; ^**^P < 0.01.

The RNA knockdown test was performed to examine the interaction between circRAB11FIP1 and proteins. Different bands were obtained (Fig. 7A). Certain proteins, such as DSC1, were found using MALDI-TOF-MS. The RNA knockdown test additionally revealed the direct binding of DSC1 with circRAB11FIP1 (Fig. 7B). The DSC1 level declined within the LV2-1- or LV2-2-transfected SKOV3 and A2780 cells compared with the cells transfected with LV2-NC. However, the DSC1 level increased in SKOV3 and A2780 cells transfected with LV6 compared with the cells transfected with LV6-NC (Fig. 7C). Certain possible DSC1 regions binding to circRAB11FIP1 were predicted by adopting catRAPID (Fig. 7D). The results found that DSC1 (△226–277 region and △351–402 region) directly bound to circRAB11FIP1 (Fig. 7E). Based on BioGRID, it was predicted that DSC1 interacted with ATG101. CircRAB11FIP1 ectopic expression or knockdown increased and decreased the interaction between DSC1 and ATG101 in A2780 and SKOV3 cells, respectively (Fig. 7F).

### CircRAB11FIP1 promoted autophagy dependent on DSC1

**Fig. 8:**
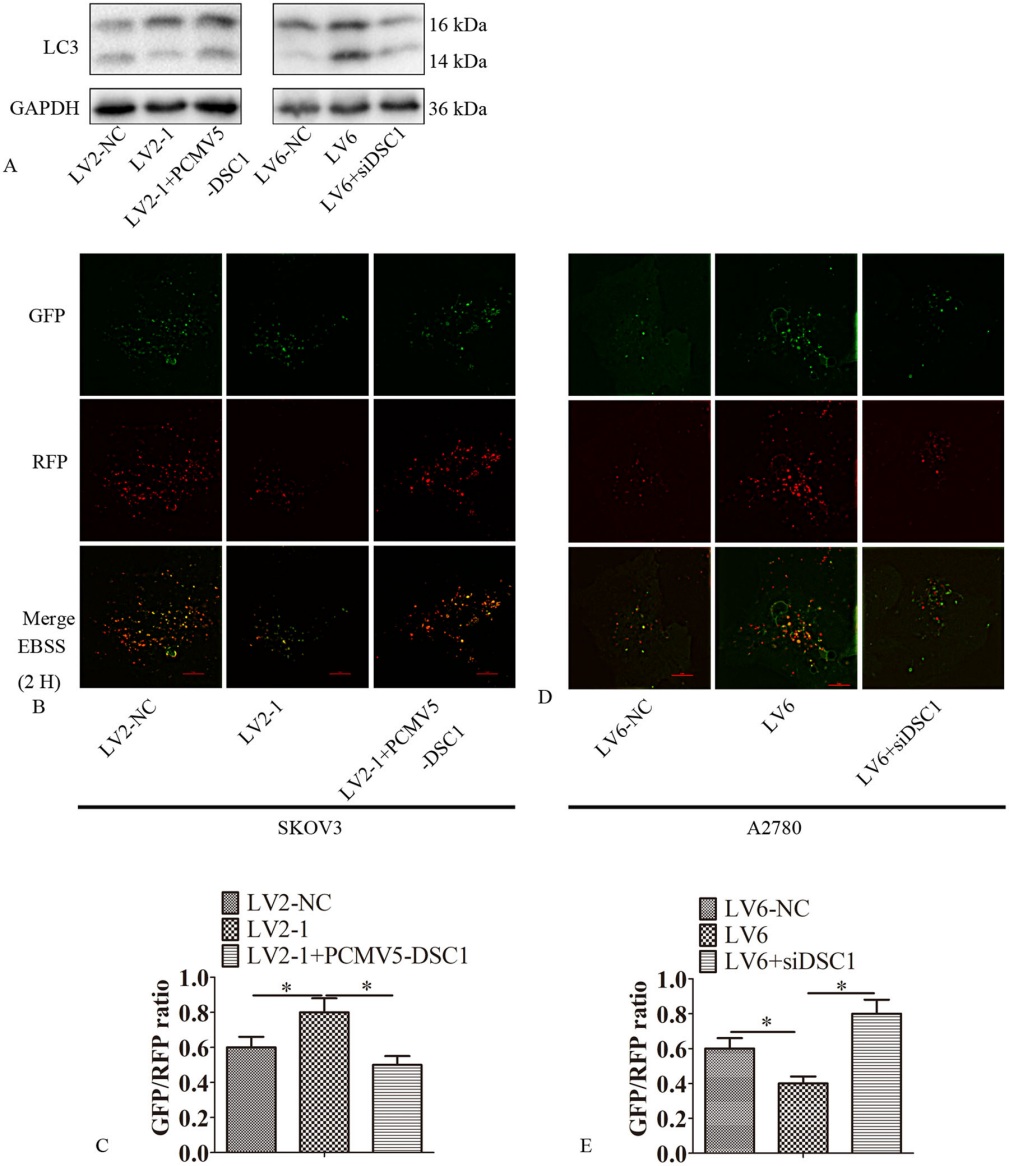
CircRAB11FIP1 enhanced the DSC1-dependent autophagy. **A** LV2-1, LV2-NC, or LV2-1 + PCMV5-DSC1 was used to transfect SKOV3 cells. LV6, LV6-NC, or LV6 + DSC1 siRNA was utilized to transfect A2780 cells. Later, LC3 expression was detected through western blot analysis. **B**, **C** LV2-1, LV2-NC, or LV2-1 + PCMV5-DSC1 was transfected into SKOV3 cells. Later, confocal microscopy was performed to analyze the distribution of mRFP-GFP-LC3 within SKOV3 cells. **D**, **E** LV6, LV6-NC, or LV6 + DSC1 siRNA was transfected into A2780 cells. Thereafter, confocal microscopy was conducted to analyze the mRFP-GFP-LC3 distribution within A2780 cells. The Image-Pro Plus 6.0 was employed for quantifying LC3 dots. Each experiment was carried out in triplicate, and typical results were presented. Error bar stands for standard error. ^*^P < 0.05; ^**^P < 0.01. Scale bar = 5 µm.

The study explored whether circRAB11FIP1 promoted autophagy dependent on DSC1. LC3-II expression decreased in LV2-1-transfected SKOV3 cells. This phenomenon was reversed after DSC1 ectopic expression. LC3-II expression elevated in A2780 cells infected with LV6. This phenomenon was partially blocked when silencing DSC1 (Figs. 8A and S3A, S3B). At the same time, the change in autophagy flux was consistent with the change in LC3-II (Fig. 8B–E).

### CircRAB11FIP1 bound to the mRNA of FTO and promoted its expression

A previous study demonstrated that N^6^-methyladenosine modification was involved in autophagy. It was predicted that the mRNA of fat mass and obesity-associated protein (FTO) might directly interact with circRAB11FIP1. The RNA pulldown assay demonstrated that circRAB11FIP1 directly bound to the mRNA of FTO (Fig. S4A). A significant enrichment of circRAB11FIP1 was observed using FTO-specific probes compared with the controls (Fig. S4B). It was predicted that circRAB11FIP1 bound to the mRNA of FTO by 14 bp (Fig. S4C). An in vivo RNA pulldown experiment with biotin-labeled FTO mRNA-specific probes was also carried out within SKOV3 cells subjected to circRAB11FIP1 transfection, either mutant or wild type (WT) with the deletion of 14 bases complementary to FTO-coding sequence (14-bp MUT). A specific enrichment of FTO mRNA was observed in both circRAB11FIP1 WT and 14-bp MUT overexpression in SKOV3 cells compared with the controls (Fig. S4D). However, circRAB11FIP1 enrichment in the mutant group was less in the WT group. The data indicated that the 14 bases were crucial for circRAB11FIP1 to combine with FTO mRNA (Fig. S4E). The FTO level also declined in LV2-1- or LV2-2-transfected SKOV3 and A2780 cells, but elevated in the LV6-transfected SKOV3 and A2780 cells (Fig. S4F–S4I).

### CircRAB11FIP1 regulated the m6A methylation of ATG5 and ATG7 mRNA via FTO

A previous study demonstrated that FTO promoted autophagy by m6A methylation of ATG5 and ATG7 at the mRNA level. CircRAB11FIP bound to the mRNA of FTO and promoted its expression. Ectopically expressed circRAB11FIP1 elevated the mRNA expression of ATG5 and ATG7, while silencing circRAB11FIP1 reduced the mRNA expression of ATG5 and ATG7. This regulation depended on FTO (Fig. S5A–S5F). MeRIP-qPCR assays found that silencing circRAB11FIP1 increased the expression of ATG5 and ATG7 m6A. Ectopically expressed circRAB11FIP1 decreased the expression of ATG5 and ATG7 m6A. This regulation was also dependent on FTO (Fig. S5G and S5H).

### Silencing circRAB11FIP1 inhibited metastasis in vivo

The study demonstrated that circRAB11FIP1 promoted the invasion of ovarian cancer cells in vitro. Hence, the study identified whether silencing circRAB11FIP1 inhibited metastasis in vivo. The results indicated that silencing circRAB11FIP1 inhibited metastasis in vivo (Fig. S6A and S6B). Also, silencing circRAB11FIP1 inhibited the expression of ATG7 and ATG14 in vivo (Fig. S6C).

## Discussion

The present study showed that the circRAB11FIP1 level was upregulated in SKOV3 cells, and it was mediated via Torn1. Meanwhile, the circRAB11FIP1 level increased in epithelial cancer tissues compared with non-carcinoma tissues. In addition, circRAB11FIP1 silencing was found to suppress the autophagic flux, which was accelerated by circRAB11FIP1 ectopic expression. The circRAB11FIP1-induced autophagy promoted ovarian cancer migration and growth. Furthermore, circRAB11FIP1 was found to sponge miR-129 to modulate ATG14 and ATG7. CircRAB11FIP1 allowed the direct binding with DSC1 to promote its level.

The study demonstrated that some circRNAs regulated autophagy^5^. CircDNMT1 promoted breast cancer progression and activated autophagy by interacting with p53 and AUF1^5^. CircHIPK3 modulated autophagy by sponging miR-124 and regulating the miR-124-STAT3 pathway. The study found that circRAB11FIP1 promoted the autophagic flux of ovarian cancer cells. A previous study showed that autophagy accelerated the metastasis of EOC^10^. The present study demonstrated that circRAB11FIP1-mediated autophagy enhanced EOC cell proliferation and migration. In addition, the circRAB11FIP1 level was upregulated in EOC tissues compared with matched non-carcinoma tissues. Therefore, circRAB11FIP1 might serve as a candidate marker to diagnose and treat EOC.

Circular RNAs were involved in cellular proliferation and invasion via sponging miRNA^11^. CircTFRC accelerated bladder carcinoma progression via sponging miR-107^12^. CircSLC8A1 suppressed malignant biological behavior by sponging miR-130b/miR-494 in bladder cancer^13^. The present study showed that circRAB11FIP1 served as the sponge for miR-129. MiR-129 inhibited the autophagy of H9c2 cells through the direct targeting of ATG14^14^. The findings suggested that miR-129 inhibited the autophagy of ovarian cancer cells by directly targeting ATG14. This was consistent with previous findings^14^. The present study also observed that miR-129 inhibited the autophagy of ovarian cancer cells by directly targeting ATG7. CircRAB11FIP1 promoted autophagy dependent on ATG7 and ATG14 via miR-129. These data indicated that circRAB11FIP1 promoted autophagy by sponging miR-129 via regulating ATG7 and ATG14.

Another mechanism by which circRNAs play a biological role is by directly binding to functional proteins^15^. CircFoxo3 interacts with p21 and CDK2 protein, promoting p21-induced CDK2 suppression and hence suppressing the progression of cell cycle in the G1 phase^16^. CircRNA cia-cGAS maintains host homeostasis by interacting with nuclear cGAS and blocking its enzymatic activity^17^. This study showed that circRAB11FIP1 promoted DSC1-dependent autophagy. Desmocollin-1 was involved in mediating cell–cell adhesion. Its expression increased in breast cancer and was associated with breast cancer cell migration and metastasis^18^. This study was novel in reporting that DSC1 was related to autophagy. The study demonstrated that ATG101 played an important role in autophagy initiation^19^. It was found that circRAB11FIP1 was bound to DSC1 to facilitate its interaction with ATG101. The data indicated that circRAB11FIP1 promoted autophagy dependent on DSC1 and ATG101.

## Conclusions

This study reported that circRAB11FIP1 expression increased in EOC. CircRAB11FIP1 promoted the autophagy and malignant behavior of EOC. CircRAB11FIP1 can serve as the possible marker for EOC diagnosis and treatment. CircRAB11FIP1 regulated the mechanism of autophagy through m6A modification and direct binding to mRNA (Fig. S7). Hence, it can serve as the possible marker for EOC diagnosis and treatment.

## Supplementary information

supplement Figure legends

Figure S1

Figure S2

Figure S3

Figure S4

Figure S5

Figure S6

Figure S7

## Data Availability

The data analyzed in this study are included in the manuscript.
