# Supplementary material for: CircRAB11FIP1 promoted autophagy flux of ovarian cancer through DSC1 and miR-129
Source: Cell Death Dis. 2021 Feb 26;12(2):219. doi: 10.1038/s41419-021-03486-1 (PMC7910449; doi:10.1038/s41419-021-03486-1)
Supplement: Supplementary file 1 — supplement Figure legends [file 41419_2021_3486_MOESM1_ESM.doc]

**Figure legends**

**Figure S1.** A regulatory network between circRNA, miRNA and mRNA constructed based on circRNA, mRNA, and miRNA results.

**Figure S2. CircRAB11FIP1 (hsa_circ_0005630) regulated the epithelial ovarian cancer cell autophagic flux.** (A) LC3 level was measured by Western blot analysis following circRAB11FIP1 silencing in A2780 cells, together with circRAB11FIP1 ectopic expression in SKOV3 cells. (B) LV6-NC or LV6 was transfected into A2780 cells, and later 3-MA or BAFA1 was added. Western blot analysis was conducted to measure the LC3 level 48 h later. (C-F) LV2-1, LV2-2, or LV2-NC was transfected into A2780 cells. LV6 or LV6-NC was transfected into SKOV3 cells. Later, confocal microscopy was performed to analyze the distribution of mRFP-GFP-LC3. Error bar stands for standard error. ^*^*P* < 0.05. Scale bar = 5 µm.

**Figure S3. CircRAB11FIP1 enhanced the DSC1-dependent autophagy.** (A and B) LV2-1, LV2-NC, or LV2-1+PCMV5-DSC1 was used to infect SKOV3 cells. LV6, LV6-NC, or LV6+DSC1 siRNA was used to infect A2780 cells. Later, LC3 expression was detected through Western blot analysis.

**Figure S4. CircRAB11FIP1 bound to the mRNA of FTO and promoted its expression.**

1. RNA pulldown was adopted for detecting the potential interaction of mRNA with circRAB11FIP1. (B) RNA knockdown was adopted for detecting the interaction of FTO mRNA with circRAB11FIP1. (C) Potential binding site between circRAB11FIP1 and FTO mRNA. (D and E) *In vivo* RNA pulldown using FTO-specific probes was performed in SKOV3 cells with circRAB11FIP1 WT or 14-bp MUT overexpression, followed by qRT-PCR to detect FTO and circRAB11FIP1. (F and G) LV2-1 and LV2-2 were transfected into A2780 and SKOV3 cells. Then, ATG2A mRNA expression was detected by qPCR. (H and I) LV6 and LV6-NC were transfected into A2780 and SKOV3 cells. Later, qPCR was conducted to detect ATG2A at the mRNA level. Each experiment was carried out in triplicate. Error bar stands for standard error. ^*^*P* < 0.05; ^**^*P* < 0.01.

**Figure S5. CircRAB11FIP1 regulated FTO to modulate the m6A methylation of ATG5 and ATG7 mRNA.**

(A and B) LV2-1 or LV2-2 was transfected into SKOV3 and A2780 cells. Later, the mRNA expression of ATG5 or ATG7 was measured using qPCR. (C-D) LV6 or LV6-NC was transfected into SKOV3 and A2780 cells. Later, the mRNA expression of ATG5 or ATG7 was measured using qPCR. (E and F) LV2-1 or LV2-2 was transfected into SKOV3 and A2780 cells. Methylated RNA immunoprecipitation (MeRIP)-qPCR analysis at the ATG5 and ATG7 mRNA m6A . (G-H) LV6 or LV6-NC was transfected into SKOV3 and A2780 cells. MeRIP-qPCR analysis at the mRNA level<s??> of ATG5 and ATG7 mRNA m6A level. The experiments were repeated three times. Error bar stands for standard error. ^*^*P* < 0.05; ^**^*P* < 0.01.

**Figure S6. CircRAB11FIP1** **regulated epithelial ovarian cancer cell invasion *in vivo*.** (A) CircRAB11FIP1 silencing inhibited the pelvic peritoneal metastasis, as found using the nude mouse model. (B) Determination of average tumor weight. (C) IHC analysis on ATG7 and ATG14. The experiments were repeated three times. The error bar stands for standard error. ^*^*P* < 0.05; ^**^*P* < 0.01. Scale bar = 100 µm.

**Figure S7. Graphical abstract of the mechanism on CircRAB11FIP1-regulated autophagy.**
